# Knowledge and attitudes toward mild traumatic brain injury among patients and family members

**DOI:** 10.3389/fpubh.2024.1349169

**Published:** 2024-05-24

**Authors:** Jian He, Zhongliang Chen, Jianjun Zhang, Xiao Liu

**Affiliations:** ^1^Department of Orthopedics, Zhejiang Hospital, Hangzhou, China; ^2^Department of Neurosurgery, Zhejiang Hospital, Hangzhou, China; ^3^Department of Radiology, Zhejiang Hospital, Hangzhou, China

**Keywords:** knowledge, attitude, traumatic brain injury, patients, cross-sectional study

## Abstract

**Introduction:**

Mild traumatic brain injury (mTBI) is a prevalent health issue with significant effects on patients' lives. Understanding and attitudes toward mTBI among patients and their families can influence management and outcomes. This study aimed to assess knowledge and attitudes toward mTBI in these groups.

**Methods:**

A cross-sectional study was conducted at Zhejiang Hospital from July 1, 2023, to September 30, 2023. Patients with mTBI and their family members participated. Data were collected via an online questionnaire covering demographic information and mTBI knowledge and attitudes. Knowledge scores ranged from 0 to 20 and attitude scores from 8 to 40. Multivariate logistic regression identified factors influencing these scores.

**Results:**

A total of 573 valid questionnaires were analyzed (289 males, 50.44%; 284 females, 49.56%). Among respondents, 258 (45.03%) had experienced a concussion. Mean knowledge and attitude scores were 11.00 ± 2.75 and 27.78 ± 4.07, respectively. Monthly per capita income of 5,000–10,000 RMB was negatively associated with knowledge and attitude scores (β = 0.160, 95% CI: [3.245 to 0.210], *P* = 0.026). Middle school education decreased the likelihood of positive attitudes toward mTBI (OR = 0.378, 95% CI: [0.1630.874], *P* = 0.023). mTBI due to falls was associated with increased likelihood of positive attitudes (OR = 3.588, 95% CI: [1.274–10.111], *P* = 0.016).

**Discussion:**

Significant gaps in knowledge and attitudes toward mTBI exist among patients and their families, influenced by income and education levels. Personal experience with mTBI from falls correlates with more positive attitudes. These findings highlight the need for targeted educational interventions to improve understanding and attitudes, ultimately enhancing patient care and management. Comprehensive, accessible mTBI education is crucial for fostering positive attitudes and better knowledge among patients and their families.

## 1 Background

Traumatic brain injury (TBI) encompasses a spectrum of severity, with mild traumatic brain injury (mTBI) or concussion being the most common subtype, constituting over 80% of TBI cases (1–3). The disruption of extensive brain networks by TBI, particularly in cases of mTBI, underscores the significant neurological effects even seemingly minor physiological disturbances can induce (4, 5). The clinical significance and long-term implications of mTBI are profound, with affected individuals potentially experiencing a wide array of consequences, including but not limited to headaches, dizziness, neck pain, drowsiness, issues with balance and vision, memory impairment, emotional and mood disturbances, fatigue, and sleep disruptions (6). As a significant global health concern, TBI is associated with elevated mortality rates, disability, and reduced health-related quality of life (7). This condition poses a serious threat to individuals' lives and has a profound effect on their overall wellbeing. Annually, over 27 million people worldwide experience TBI (8), frequently resulting in cognitive and functional deficits, impeding daily life activities, and diminishing overall quality of life (9–11). Therefore, a comprehensive approach is essential to address TBI, with a specific focus on mTBI, as it is a common contributor to neurological morbidity and a leading cause of disability, adversely affecting patient function and quality of life.

In accordance with the Knowledge, Attitude, and Practice (KAP) model, individual behaviors are influenced by their knowledge and attitude (12). Knowledge can be considered as a distal determinant of behavior, which is mediated by attitude. Knowledge holds particular significance in shaping attitudes toward health-related behaviors within the framework of the knowledge-attitude-behavior model (13). Given that patients and their family members are either directly affected by or closely connected to individuals with mTBI, their levels of knowledge and attitudes assume a pivotal role in the processes of rehabilitation and management. Gaining insights into their perspectives and needs can greatly aid healthcare professionals in better fulfilling their requirements and delivering more effective medical care. Currently, there is a notable absence of pertinent research within this specific domain, underscoring an existing gap in the academic literature.

Therefore, this study aimed to investigate the knowledge and attitudes toward mTBI among patients and their family members.

## 2 Methods

### 2.1 Study design and participants

This cross-sectional study was conducted between July 1, 2023 and September 30, 2023 at Zhejiang Hospital among patients and their family members of mTBI. This study received ethical approval from the Medical Ethics Committee of Zhejiang Hospital and obtained informed consent from all participants. Inclusion Criteria: (1) Patients with mild traumatic brain injury/Family members of patients with mild traumatic brain injury; (2) History of mild traumatic brain injury within the past year. Mild TBI (mTBI) is defined as the initial Glasgow coma score (GCS) of 13–15, loss of consciousness (LOC) of <30 min, duration of change in mental state (AOC), and posttraumatic amnesia (PTA) of <24 h after external violence on the patient's head, with a negative head CT scan result (14). Exclusion criteria included: (1) Questionnaire completion time of <90 s, suggesting insufficient engagement; (2) Participants expressing disagreement in their responses, indicating potential misunderstandings; (3) Incorrect answers to trap questions designed to test the accuracy of knowledge. Informed consent was obtained from all participants.

### 2.2 Questionnaire and quality control

The questionnaire was developed based on the 2022 Neurosurgery Guidelines for Diagnosis and Treatment of Cranial Injuries and previous literatures (15–17). A pilot test was conducted involving a small sample of 22 respondents. Participants were recruited through a randomized questionnaire survey administered by neurosurgeons, targeting patients with mild brain injuries and their families at neurosurgery outpatient and inpatient clinics. The internal consistency of the questionnaire was assessed using Cronbach's α coefficient, which yielded a value of 0.856, indicating satisfactory internal reliability. The final questionnaire comprised four sections: demographic information (covering gender, age, education, and professional title), a knowledge section, and an attitude section. The knowledge segment encompassed 13 questions, with the 9th and 10th questions each containing 6 and 3 sub-questions, respectively. Respondents received 1 point for correct answers and 0 points for incorrect or unclear responses, resulting in a knowledge score range of 0–20 points. The attitude section consisted of eight questions, employing a five-point Likert scale ranging from very positive (five points) to very negative (one point), with a total score range of 8–40 points. Positive attitude was defined as achieving scores surpassing 70% of the maximum possible score in each respective section (18–20).

Online e-questionnaires were created using the Wen-Juan-Xing online platform in China (https://www.wjx.cn/app/survey.aspx), and QR codes were provided for access to the electronic version. The distribution of questionnaires occurred in emergency surgery, neurosurgery, and orthopedic consultation rooms, as well as during follow-up visits and appointments with patients recovering from cerebral concussion, facilitated by psychiatrists and orthopedic doctors. To ensure the quality and completeness of the questionnaire results, each IP address could only be used once for submission, and every question was mandatory. The research team meticulously checked all questionnaires for completeness, consistency, and validity.

### 2.3 Statistical analysis

Data analysis was conducted using SPSS 26.0 (IBM, Armonk, NY, USA). Continuous data are presented as means and standard deviations (SD), while categorical data are expressed as *n* (%). Continuous variables underwent a normality test, with the *t*-test for normally distributed data and the Wilcoxon Mann–Whitney test for non-normally distributed data when comparing two groups. For three or more groups with normally distributed continuous variables and uniform variance, ANOVA was used for comparisons, while the Kruskal–Wallis test was employed for non-normally distributed data. Multivariate analysis was used to explore the factors influencing knowledge and attitudes score and positive attitudes. A two-sided *P*-value <0.05 was considered statistically significant.

## 3 Results

Initially, a total of 950 questionnaires were collected in the study, excluding 12 cases of questionnaires with too short response time (<90 s); two cases of disagreement with participation; and 363 cases of wrong answers to the trap questions of the knowledge dimensions, leaving 573 valid questionnaires with an effective rate of 60.25%. One of the questionnaires was not filled in for age, which was supplemented by using the mean value. Among them, 289 (50.44%) were males, 312 (54.45%) were aged 18–40 years, 203 (35.43%) had junior college or undergraduate education. Two hundred-eighteen (38.05%) suffered from mTBI due to external collision such as a car accident, and 258 (45.03%) experienced once of concussion.

The mean knowledge and attitude scores were 11.00 ± 2.75 (possible range: 0–20) and 27.78 ± 4.07 (possible range: 8–40), respectively. The knowledge score varied from patients with different monthly per capita income (*P* = 0.029) and the reasons they suffered a mTBI (*P* = 0.007). As for the attitude score, there were difference among patients with different age (*P* < 0.001), education (*P* = 0.007), and the reasons they suffered a mTBI (*P* < 0.001; Table 1).

**Table 1:** Baseline characteristics and KA scores.

	***N* (%)**	**Knowledge, mean ±SD**	***P*-value**	**Attitude, mean ±SD**	***P*-value**
*N* = 573				
Total score		11.00 ± 2.75		27.78 ± 4.07	
**Gender**			0.988		0.607
Male	289 (50.44)	11.00 ± 2.48		27.87 ± 3.74	
Female	284 (49.56)	11.00 ± 2.99		27.69 ± 4.38	
**Age, years**			0.656		<0.001
18–40	312 (54.45)	10.93 ± 2.94		28.23 ± 4.08	
41–59	257 (44.85)	11.10 ± 2.38		27.15 ± 3.93	
60 and above	4 (0.70)	10.25 ± 7.14		33.25 ± 4.50	
**Residence**			0.151		0.585
Rural	93 (16.23)	10.62 ± 2.90		27.99 ± 4.23	
Urban	480 (83.77)	11.07 ± 2.71		27.74 ± 4.04	
**Education**			0.136		0.007
Primary school and below	58 (10.12)	11.45 ± 2.13		27.43 ± 3.73	
Middle school	159 (27.75)	11.04 ± 2.44		27.18 ± 3.74	
High school/technical secondary school	72 (12.57)	11.17 ± 2.77		27.04 ± 3.74	
Junior college/undergraduate	203 (35.43)	10.63 ± 3.18		28.58 ± 4.30	
Postgraduate and above	81 (14.14)	11.37 ± 2.42		27.85 ± 4.36	
**Medical-related occupation**			0.071		0.100
Yes	2 (0.35)	14.50 ± 2.12		32.50 ± 2.12	
No	571 (99.65)	10.99 ± 2.74		27.76 ± 4.07	
**Monthly per capita income, RMB**			0.029		0.063
<2,000	56 (9.77)	11.36 ± 2.13		27.89 ± 3.27	
2,000–5,000	168 (29.32)	11.27 ± 2.50		27.35 ± 3.45	
5,000–10,000	173 (30.19)	10.47 ± 3.07		27.42 ± 4.33	
10,000–20,000	120 (20.94)	10.99 ± 2.57		28.43 ± 4.22	
>20,000	56 (9.77)	11.48 ± 3.09		28.66 ± 5.04	
**Marital status**			0.554		0.893
Unmarried	94 (16.40)	10.72 ± 2.87		27.84 ± 3.76	
Married	322 (56.20)	11.11 ± 2.83		27.86 ± 4.54	
Divorced	106 (18.50)	10.82 ± 2.45		27.63 ± 3.30	
Widowed	51 (8.90)	11.14 ± 2.57		27.45 ± 2.77	
**Reason of mTBI**			0.007		<0.001
Sports	148 (25.83)	11.01 ± 2.42		27.64 ± 4.04	
External collisions, such as a car accident	218 (38.05)	11.29 ± 2.51		27.53 ± 3.78	
Falling from a height or other	21 (3.66)	9.19 ± 5.01		31.43 ± 5.51	
Accidental falls	186 (32.46)	10.85 ± 2.84		27.77 ± 4.07	
**Times of concussion experienced by you (or patients in your household)**			0.365		0.278
Once	258 (45.03)	11.03 ± 2.59		27.93 ± 3.86	
Twice	140 (24.43)	11.10 ± 2.77		27.69 ± 3.65	
3 or more	84 (14.66)	11.21 ± 2.43		27.06 ± 4.28	
Unclear	91 (15.88)	10.55 ± 3.34		28.16 ± 4.95	

The distribution of knowledge dimensions revealed that the three knowledge items with the highest correctness rates were as follows: “*Concussion is also known as mTBI*.” (K1) with 82.90%, “*mTBI is more severe than a concussion*.” (K7) with 82.90%, and “*Individuals who have not yet recovered from a concussion should avoid further head impacts*.” (K12) with 71.03%. Contrarily, the three items with the lowest correctness rates were “*Common psychological symptoms in concussion patients include nightmares*.” (K10.3) with 16.93%, “*Patients with concussion typically do not require specialized treatment*.” (K11) with 19.72%, and “*One of the methods for assessing the recovery progress after a concussion is to inquire about the patient's feelings*.” (K13) with 28.97% (Table 2).

**Table 2:** Responses in the knowledge dimensions.

**Items, *n* (%)**	**Correctness rate**
1. Concussion is also known as mTBI	475 (82.90)
2. Altered consciousness in concussion includes conditions such as mental confusion, amnesia, or loss of consciousness	325 (56.72)
3. After head trauma, a brief period of unconsciousness may occur immediately, with consciousness often returning within 30 min	336 (58.64)
4. Concussion can potentially result in brain injury even if a person does not lose consciousness	332 (57.94)
5. It is necessary for concussion patients to experience a loss of consciousness	388 (67.71)
6. Individuals who have suffered a concussion may have difficulty recalling events that occurred before the concussion	320 (55.85)
7. MTBI is more severe than a concussion	475 (82.90)
8. The use of cranial computed tomography scans or magnetic resonance imaging can determine if brain injury has occurred due to a concussion	318 (55.50)
9. **Characteristics of concussion include:**	
9.1. Purposeless staring or unclear speech	348 (60.73)
9.2. Delayed language and motor responses	351 (61.26)
9.3. Easily distracted attention and an inability to concentrate	324 (56.54)
9.4. Difficulty discerning date, time, and location	266 (46.42)
9.5. Motor incoordination: stumbling while walking	328 (57.24)
9.6. Memory impairment: repeating the same question	289 (50.44)
10. **Common psychological symptoms in concussion patients include:**	
10.1. Irritability or easy anger	309 (53.93)
10.2. Sadness or depression	335 (58.46)
10.3. Nightmares	97 (16.93)
11. Patients with concussion typically do not require specialized treatment	113 (19.72)
12. Individuals who have not yet recovered from a concussion should avoid further head impacts	407 (71.03)
13. One of the methods for assessing the recovery progress after a concussion is to inquire about the patient's feelings	166 (28.97)

Participants' attitudes toward mTBI (concussion) varied considerably, 68.76% of respondents recognized the necessity of seeking immediate medical consultation when symptoms of a concussion are present (A1). Additionally, 67.36% stressed the importance of closely monitoring the condition following a concussion to prevent deterioration (A2). 66.97% believed that even in the absence of apparent symptoms after an external head collision, it is essential to rule out the possibility of a concussion (A3). Moreover, 65.45% acknowledged the importance of monitoring memory, either for themselves or patients in their household, following a concussion diagnosis (A4). When it came to concerns about concussion recovery (A5), 67.89% expressed varying degrees of concern, while 9.95% believed in generally good recovery and considered excessive worry unnecessary. Regarding emotional changes and attention, 68.24% agreed on the need to promptly address emotional changes for oneself or patients (A6). Conversely, only 10.65% believed that short-term memory loss symptoms following a concussion are normal, while 60.74% expressed some level of concern (A7). Furthermore, 65.79% recognized that severe symptoms resulting from a concussion could lead to consequences such as death (A8; Table 3).

**Table 3:** Responses in the attitude dimensions.

**Items, *n* (%)**	**Strongly agree**	**Agree**	**Neutral**	**Disagree**	**Strongly disagree**
1. Immediate medical consultation is necessary when symptoms of concussion are present	261 (45.55)	133 (23.21)	138 (24.08)	21 (3.66)	20 (3.49)
2. Close observation of the condition following a concussion is necessary to prevent deterioration	177 (30.89)	209 (36.47)	133 (23.21)	24 (4.19)	30 (5.24)
3. Even without apparent symptoms after an external head collision, the possibility of a concussion should be ruled out	229 (39.97)	149 (26.00)	159 (27.75)	16 (2.79)	20 (3.49)
4. Following a concussion diagnosis, it is important to monitor memory for yourself or patients in your household	159 (27.75)	216 (37.70)	146 (25.48)	26 (4.54)	26 (4.54)
5. Recovery from a concussion is generally good, and excessive worry is unnecessary	29 (5.06)	28 (4.89)	127 (22.16)	144 (25.13)	245 (42.76)
6. Prompt attention to emotional changes for yourself or patients is necessary	246 (42.93)	145 (25.31)	139 (24.26)	22 (3.84)	21 (3.66)
7. Short-term memory loss symptoms following a concussion are normal, and excessive worry is unnecessary	35 (6.11)	26 (4.54)	164 (28.62)	234 (40.84)	114 (19.90)
8. Severe symptoms in a concussion can lead to consequences such as death	135 (23.56)	242 (42.23)	140 (24.43)	26 (4.54)	30 (5.24)

Multivariate analysis showed that monthly per capita income between 5,000 and 10,000 RMB was independently associated with knowledge and attitude score [β = −0.160, 95% CI: (−3.245 to 0.210), *P* = 0.026; Table 4]. Moreover, middle school education [OR = 0.378 95% CI: (0.163–0.874), *P* = 0.023] and suffering a mTBI due to falling from a height or other [OR = 3.588, 95% CI: (1.274–10.111), *P* = 0.016] were independently associated with positive attitude (Table 5).

**Table 4:** Analysis of factors affecting KA scores.

**Variables**	**Univariate analysis**		**Multivariate analysis**	
	**β (95%CI)**	***P*-value**	**β (95% CI)**	***P*-value**
**Gender**				
Male	0.018 (−0.635 to 0.992)	0.667		
**Age**				
18–40	−0.437 (−9.217 to 0.525)	0.080	−0.313 (−8.264 to 2.044)	0.236
41–59	−0.528 (−10.132 to −0.378)	0.035	−0.454 (−9.794 to 0.757)	0.093
**Residence**				
Rural	−0.015 (−1.299 to 0.908)	0.728		
**Education**				
Middle school	−0.060 (−2.151 to 0.833)	0.386		
High school/technical secondary school	−0.045 (−2.387 to 1.045)	0.443		
Junior college/undergraduate	0.032 (−1.120 to 1.775)	0.657		
Postgraduate and above	0.024 (−1.330 to 2.016)	0.687		
**Medical-related occupation**				
Yes	0.098 (1.384 to 15.120)	0.019	0.066 (−1.766 to 12.840)	0.137
**Monthly per capita income**				
<2,000	−0.054 (−2.719 to 0.933)	0.337	0.036 (−1.546 to 2.736)	0.585
2,000–5,000	−0.140 (−3.015 to −0.033)	0.045	−0.019 (−2.008 to 1.585)	0.817
5,000–10,000	−0.209 (−3.738 to −0.767)	0.003	−0.160 (−3.245 to −0.210)	0.026
10,000–20,000	−0.060 (−2.290 to 0.837)	0.362	−0.047 (−2.132 to 0.981)	0.468
**Marital status**				
Married	0.041 (−0.732 to 1.554)	0.480		
Divorced	−0.009 (−1.492 to 1.270)	0.875		
Widowed	0.001 (−1.671 to 1.720)	0.977		
**Reason of mTBI**				
External collisions, such as a car accident	0.016 (−0.875 to 1.198)	0.760		
Falling from a height or other	0.075 (−0.306 to 4.233)	0.090		
Accidental falls	−0.004 (−1.109 to 1.035)	0.946		
**Times of concussion experienced by you (or patients in your household)**				
Twice	−0.015 (−1.195 to 0.852)	0.742		
3 or more	−0.049 (−1.908 to 0.541)	0.273		
Unclear	−0.018 (−1.432 to 0.946)	0.688		

The reference categories for each variable are as follows: Gender (female), Age (60 and above), Residence (urban), Education (primary school and below), Medical-related occupation (no), Monthly per capita income (>20,000), Marital status (unmarried), reason of mTBI (sports), Times of concussion experienced (once).

**Table 5:** Analysis of factors affecting positive attitudes.

**Variables**	**Univariate**		**Multivariate**	
	**OR (95%CI)**	***P*-value**	**OR (95%CI)**	***P*-value**
Knowledge score	0.989 (0.918–1.065)	0.767		
**Gender**				
Male	1.044 (0.694–1.572)	0.835		
**Age**				
18–40	0.109 (0.011–1.065)	0.057	0.123 (0.010–1.512)	0.102
41–59	0.053 (0.005–0.520)	0.012	0.111 (0.009–1.405)	0.090
**Residence**				
Rural	1.487 (0.886–2.495)	0.133		
**Education**				
Middle school	0.387 (0.173–0.866)	0.021	0.378 (0.163–0.874)	0.023
High school/technical secondary school	0.433 (0.166–1.130)	0.087	0.514 (0.170–1.561)	0.240
Junior college/undergraduate	1.351 (0.679–2.691)	0.392	0.983 (0.233–4.143)	0.982
Postgraduate and above	1.212 (0.549–2.676)	0.635	0.888 (0.192–4.109)	0.879
**Medical-related occupation**				
Yes	/	0.999		
**Monthly per capita income**				
<2,000	0.561 (0.235–1.340)	0.193	1.189 (0.310–4.552)	0.801
2,000–5,000	0.310 (0.148–0.648)	0.002	0.624 (0.187–2.084)	0.444
5,000–10,000	0.501 (0.251–0.998)	0.049	0.610 (0.287–1.294)	0.198
10,000–20,000	0.983 (0.493–1.961)	0.962	1.058 (0.516–2.170)	0.878
**Marital status**				
Married	0.811 (0.472–1.394)	0.449		
Divorced	0.631 (0.316–1.261)	0.193		
Widowed	0.491 (0.195–1.240)	0.132		
**Reason of mTBI**				
External collisions, such as a car accident	0.911 (0.539–1.541)	0.728	1.063 (0.617–1.832)	0.826
Falling from a height or other	4.327 (1.681–11.137)	0.002	3.588 (1.274–10.111)	0.016
Accidental falls	0.848 (0.490–1.470)	0.558	0.918 (0.520–1.622)	0.768
**Times of concussion experienced by you (or patients in your household)**				
Twice	0.688 (0.399–1.186)	0.178		
3 or more	0.802 (0.426–1.511)	0.495		
Unclear	1.248 (0.714–2.183)	0.436		

The reference categories for each variable are as follows: Gender (female), Age (60 and above), Residence (urban), Education (primary school and below), Medical-related occupation (no), Monthly per capita income (>20,000), Marital status (unmarried), Reason of mTBI (sports), Times of concussion experienced (once).

## 4 Discussion

As evidenced by our findings, patients and family members exhibit inadequate knowledge and suboptimal attitudes toward mTBI, underscoring the critical need for healthcare professionals across various settings—from emergency surgery units to outpatient psychiatric clinics—to enhance clinical practices. This enhancement should particularly focus on patient and carer education, adapted to the specific contexts of different healthcare environments. Given the varied causes of mTBI, from sports-related concussion (SRC) to falls from a height, our study advocates for personalized support that addresses the unique challenges and needs associated with each type of injury. This approach is grounded in emerging research that supports tailored interventions based on the underlying cause of mTBI to improve patient outcomes. Beyond merely listing examples, our findings illuminate the profound implications of enhancing mTBI awareness and management. Specifically, by addressing the knowledge and attitude gaps identified, healthcare practices can evolve to more effectively educate patients and their families—especially those from lower-income backgrounds—about mTBI. Such informed engagement is crucial for improving mTBI outcomes and patient adherence to recommended care plans, thereby facilitating more effective management of mTBI.

The study findings indicate that patients and their family members exhibit inadequate knowledge and less favorable attitudes toward mTBI. These results are in line with previous studies, which has consistently highlighted a general lack of awareness and suboptimal attitudes toward TBI in healthcare settings (21, 22). The study also identifies several influencing factors, such as income, education, and the causes of TBI, which should be considered in clinical practice. For instance, socioeconomic status, as indicated by monthly per capita income, plays a significant role in knowledge and attitude scores, suggesting that tailored interventions may be necessary for individuals with lower incomes, echoing findings from studies in other health domains (23). Furthermore, education level demonstrated a significant association with attitudes toward mTBI, where individuals with higher educational backgrounds, particularly those with junior college/undergraduate degrees, exhibited more positive attitudes. This finding underscore that education not only enhances knowledge but also positively influences health-related attitudes, potentially leading to more effective coping strategies and adherence to treatment protocols in the face of health challenges. The reasons behind TBI occurrences, such as falls from heights, are associated with more positive attitudes, emphasizing the need for personalized support based on the circumstances of the injury. Additionally, variations in attitude scores among patients of different age groups and educational backgrounds highlight the importance of targeted educational initiatives. In light of these findings, healthcare professionals should prioritize patient and family education, particularly for those from diverse demographic backgrounds, to enhance clinical management and outcomes related to mTBI (24, 25).

The findings from the knowledge dimension of this study provide insight into the existing deficiencies in understanding mTBI and concussion among patients and their family members. While some respondents exhibited a relatively accurate understanding of certain aspects, such as the definition of concussion and its potential consequences, a significant proportion displayed gaps in knowledge. For instance, misconceptions regarding the necessity of experiencing loss of consciousness or the severity of mTBI compared to a concussion were prevalent. The assessment of recovery progress after a concussion was also found to be poorly understood, as a relatively low percentage of respondents recognized the importance of assessing patients' feelings. Such knowledge gaps underline the critical need for targeted education and awareness campaigns. To improve clinical practice, initiatives should focus on disseminating accurate information about the nature of mTBI, the importance of assessing recovery, and the potential psychological symptoms (26, 27). Additionally, promoting the understanding that individuals with concussion should avoid further head impacts and that specialized treatment might be necessary for some patients is essential for enhancing the management and outcomes of mTBI cases (28, 29). For clinicians in emergency settings, this might mean emphasizing the seriousness of any head injury, regardless of immediate symptoms. In contrast, those in rehabilitation or psychiatric settings might focus on educating patients about the recovery timeline and potential psychological symptoms.

The results from the attitude dimension of this study reveal a spectrum of attitudes held by patients and their family members toward mTBI and concussion. While a notable proportion of respondents demonstrated a positive attitude by recognizing the importance of immediate medical consultation when TBI symptoms are present, and the need for close observation following a concussion, there were also prevalent neutral and negative attitudes in certain aspects. Notably, a substantial number of respondents held neutral or negative attitudes regarding the necessity of excessive worry and emotional changes following a concussion, and the potential severity of symptoms, including the possibility of death. These findings are consistent with previous studies indicating variations in attitudes toward TBI and the associated emotional and cognitive aspects (30). To address these deficiencies in attitudes, initiatives to improve clinical practice should aim at fostering a more informed and empathetic perspective. Education and counseling should be directed toward emphasizing the importance of timely medical attention, vigilant observation, and supportive emotional care following a concussion (31, 32). Initiatives should also work to dispel misconceptions regarding symptom severity and emphasize the potential consequences of severe TBI (33, 34). Ultimately, by addressing the specific knowledge gaps and misconceptions identified in our study, these efforts are expected to foster a more positive and well-informed attitude among patients and their families. This direct targeting of educational content, informed by our findings, is anticipated to lead to better clinical management and outcomes for mTBI cases (35, 36).

This study has several limitations. First, data were collected through online questionnaires, which may introduce selection bias, as it excludes individuals without internet access or those who choose not to participate. Second, the study relies on self-reported information, which can be subject to recall bias and social desirability bias, potentially affecting the accuracy of the responses. Additionally, the study's cross-sectional design limits the ability to establish causality or assess changes in knowledge and attitudes over time. Furthermore, the study was conducted in a specific geographic area, which may limit the generalizability of the findings to other regions or populations with different socio-cultural backgrounds. The recruitment strategy utilized in this study, spanning diverse healthcare settings and periods, enriches the applicability of our findings. However, it also introduces considerations for future research, particularly in examining how seasonal variations and different healthcare environments may impact the prevalence and understanding of mTBI. Such insights could inform more nuanced approaches to patient education and care, potentially tailoring strategies to specific times of the year or patient populations.

In conclusion, patients and family members had inadequate knowledge and suboptimal attitude toward mTBI. Special attention should be given to individuals with lower monthly per capita incomes, as they exhibited less knowledge and less favorable attitudes. Tailored interventions and support programs should be developed to address the specific needs of this demographic. Additionally, recognizing the influence of injury causes on attitudes, such as falling from a height, can guide clinicians in providing personalized counseling and support.

## Data availability statement

The original contributions presented in the study are included in the article/Supplementary material, further inquiries can be directed to the corresponding authors.

## Ethics statement

The studies involving humans were approved by Ethics Review Committee of Zhejiang Hospital [2022-139K]. The studies were conducted in accordance with the local legislation and institutional requirements. Written informed consent for participation was not required from the participants or the participants' legal guardians/next of kin in accordance with the national legislation and institutional requirements.

## Author contributions

JH: Conceptualization, Data curation, Formal analysis, Investigation, Writing – original draft. ZC: Conceptualization, Data curation, Formal analysis, Investigation, Writing – original draft. JZ: Conceptualization, Formal analysis, Writing – original draft, Writing – review & editing. XL: Conceptualization, Data curation, Formal analysis, Investigation, Writing – original draft.
