# Supplementary material for: Knowledge and attitudes toward mild traumatic brain injury among patients and family members
Source: Front Public Health. 2024 May 24;12:1349169. doi: 10.3389/fpubh.2024.1349169 (PMC11157019; doi:10.3389/fpubh.2024.1349169)
Supplement: Supplementary file 1 [file Table_1.DOCX]

| **Knowledge and Attitudes toward Mild Traumatic Brain Injury among Patients and Family Members** | | | |
| --- | --- | --- | --- |
| **Part I Basic Information** | | | |
| **1.** **Gender** | | a. Male  b. Female |  |
| **2.Your age** | | a. 18-40  b. 41-59  c. 60 and above | |
| **3.** **Residence** | | a. Rural  b. Urban | |
| **4.** **Education** | | a. Primary school and below  b. Middle school  c. High School/Technical secondary school  d. Junior college/Undergraduate  e. Postgraduate and above | |
| **5.** **Medical-related occupation** | | a. Yes  b. No | |
| **6.** **Monthly per capita income, yuan** | | a. < 2000  b. 2000-5000  c. 5000-10000  d. 10,000-20,000  e. >20,000 | |
| **8.** **Marital status** | | a. Unmarried  b. Married  c. Divorced  d. Widowed | |
| **9.** **Reason of mTBI** | | a. Sports  b. External collisions, such as a car accident  c. Falling from a height or other  d. Accidental falls | |
| **10.** **Times of concussions experienced by you (or patients in your household)** | | a. Once  b. Twice  c. 3 or more  d. Unclear | |

**Part II Knowledge**

| **1. Concussion is also known as mTBI.** | a. Correct | b. Wrong | c. Unclear |
| --- | --- | --- | --- |
| **2. Altered consciousness in concussion includes conditions such as mental confusion, amnesia, or loss of consciousness.** | a. Correct | b. Wrong | c. Unclear |
| **3. After head trauma, a brief period of unconsciousness may occur immediately, with consciousness often returning within 30 minutes.** | a. Correct | b. Wrong | c. Unclear |
| **4. Concussion can potentially result in brain injury even if a person does not lose consciousness.** | a. Correct | b. Wrong | c. Unclear |
| **5. It is necessary for concussion patients to experience a loss of consciousness.** | a. Correct | b. Wrong | c. Unclear |
| **6. Individuals who have suffered a concussion may have difficulty recalling events that occurred before the concussion.** | a. Correct | b. Wrong | c. Unclear |
| **7. MTBI is more severe than a concussion.** | a. Correct | b. Wrong | c. Unclear |
| **8. The use of cranial computed tomography scans or magnetic resonance imaging can determine if brain injury has occurred due to a concussion.** | a. Correct | b. Wrong | c. Unclear |
| **9. Characteristics of concussion include:** |  |  |  |
| **9.1 Purposeless staring or unclear speech** | a. Correct | b. Wrong | c. Unclear |
| **9.2 Delayed language and motor responses** | a. Correct | b. Wrong | c. Unclear |
| **9.3 Easily distracted attention and an inability to concentrate** | a. Correct | b. Wrong | c. Unclear |
| **9.4 Difficulty discerning date, time, and location** | a. Correct | b. Wrong | c. Unclear |
| **9.5 Motor incoordination: stumbling while walking** | a. Correct | b. Wrong | c. Unclear |
| **9.6 Memory impairment: repeating the same question** | a. Correct | b. Wrong | c. Unclear |
| **10. Common psychological symptoms in concussion patients include:** |  |  |  |
| **10.1 Irritability or easy anger** | a. Correct | b. Wrong | c. Unclear |
| **10.2 Sadness or depression** | a. Correct | b. Wrong | c. Unclear |
| **10.3 Nightmares** | a. Correct | b. Wrong | c. Unclear |
| **11. Patients with concussion typically do not require specialized treatment.** | a. Correct | b. Wrong | c. Unclear |
| **12. Individuals who have not yet recovered from a concussion should avoid further head impacts.** | a. Correct | b. Wrong | c. Unclear |
| **13. One of the methods for assessing the recovery progress after a concussion is to inquire about the patient's feelings.** | a. Correct | b. Wrong | c. Unclear |

**Part III Attitude**

| **1. Immediate medical consultation is necessary when symptoms of concussion are present.** | a. Strongly agree | b. Agree | c. Neutral | d. Disagree | e. Strongly disagree |
| --- | --- | --- | --- | --- | --- |
| **2. Close observation of the condition following a concussion is necessary to prevent deterioration.** | a. Strongly agree | b. Agree | c. Neutral | d. Disagree | e. Strongly disagree |
| **3. Even without apparent symptoms after an external head collision, the possibility of a concussion should be ruled out.** | a. Strongly agree | b. Agree | c. Neutral | d. Disagree | e. Strongly disagree |
| **4. Following a concussion diagnosis, it is important to monitor memory for yourself or patients in your household.** | a. Strongly agree | b. Agree | c. Neutral | d. Disagree | e. Strongly disagree |
| **5. Recovery from a concussion is generally good, and excessive worry is unnecessary.** | a. Strongly agree | b. Agree | c. Neutral | d. Disagree | e. Strongly disagree |
| **6. Prompt attention to emotional changes for yourself or patients is necessary.** | a. Strongly agree | b. Agree | c. Neutral | d. Disagree | e. Strongly disagree |
| **7. Short-term memory loss symptoms following a concussion are normal, and excessive worry is unnecessary.** | a. Strongly agree | b. Agree | c. Neutral | d. Disagree | e. Strongly disagree |
| **8. Severe symptoms in a concussion can lead to consequences such as death.** | a. Strongly agree | b. Agree | c. Neutral | d. Disagree | e. Strongly disagree |
